# Adenosine heteroreceptor complexes in the basal ganglia are implicated in Parkinson’s disease and its treatment

**DOI:** 10.1007/s00702-019-01969-2

**Published:** 2019-01-14

**Authors:** Dasiel O. Borroto-Escuela, Kjell Fuxe

**Affiliations:** 1 Department of Neuroscience, Karolinska Institutet, Biomedicum, B0851, Solnavägen 9, 17177 Stockholm, Sweden; 2 Observatorio Cubano de Neurociencias, Grupo Bohío-Estudio, Zayas 50, 62100 Yaguajay, Cuba

**Keywords:** G protein-coupled receptor, Neurodegeneration, Adenosine receptor, Heteroreceptor complexes, Oligomerization, Parkinson’s diseases, Basal ganglia

## Abstract

The adenosine homo, iso and heteroreceptor complexes in the basal ganglia play a highly significant role in modulating the indirect and direct pathways and the striosomal projections to the nigro-striatal DA system. The major adenosine receptor complexes in the striato-pallidal GABA neurons can be the A2AR–D2R and A2AR–D2R–mGluR5 receptor complexes, in which A2AR protomers and mGluR5 protomers can allosterically interact to inhibit D2R protomer signaling. Through a reorganization of these heteroreceptor complexes upon chronic dopaminergic treatment a pathological and prolonged inhibition of D2R receptor protomer signaling can develop with motor inhibition and wearing off of the therapeutic effects of levodopa and dopamine receptor agonists. The direct pathway is enriched in D1R in and around glutamate synapses enhancing the ability of these GABA neurons to be activated and increase motor initiation. The brake on these GABA neurons is in this case exerted by A1R forming A1R–D1R heteroreceptor complexes in which they allosterically inhibit D1R signaling and thereby reduce motor initiation. Upon chronic levodopa treatment a reorganization of the D1R heteroreceptor complexes develops with the formation of putative A1R–D1R–D3 in addition to D1R–D3R complexes in which D3R enhances D1R protomer signaling and may make the A1R protomer brake less effective. Alpha-synuclein monomers–dimers are postulated to form complexes with A2AR homo and heteroprotomers in the plasma membrane enhancing alpha-synuclein aggregation and toxicity. The alpha-synuclein fibrils formed in the A2AR enriched dendritic spines of the striato-pallidal GABA neurons may reach the surrounding DA terminals via extracellular-vesicle-mediated volume transmission involving internalization of the vesicles and their cargo (alpha-synuclein fibrils) into the vulnerable DA terminals, enhancing their degeneration followed by retrograde flow of these fibrils in the DA axons to the vulnerable nigral DA nerve cells.

## Introduction to the field of homo-and heteroreceptor complexes

There exists substantial evidence for the existence of G protein-coupled receptor (GPCR) homo and heteroreceptor complexes with allosteric receptor–receptor interactions in the central nervous system (CNS) (Borroto-Escuela et al. 2014a, 2017a; Franco et al. 2016; Fuxe et al. 1983, 2014a, 2010b; Guo et al. 2008; Lee et al. 2002; Liu et al. 2000; Marshall et al. 1999; Milligan 2013). Through the receptor heteromerization the allosteric receptor–receptor interactions can develop and produce alterations in recognition including novel allosteric binding sites, pharmacology, signaling, and trafficking of the participating receptors (receptor protomers). This leads to biased and diverse signaling of the receptor heteromer signaling and to a specific integrated response at the molecular level (Fuxe et al. 2014e). In tissues, e.g., the brain, the terms homo and heteroreceptor complexes are instead used since the receptors also bind to a substantial number of adapter proteins many of which remain to be identified. There is also lack of knowledge on the stoichiometry of the participating receptor protomers in GPCR heteroreceptor complexes. However, to-day super-resolution imaging methods (Owen et al. 2013) and spatial intensity distribution analysis (Ward et al. 2015) have been developed which can be used to determine the stoichiometry in cellular models. The GPCR complexes can also contain ion channel receptors, receptor tyrosine kinases (RTKs), sets of G protein interacting proteins and/or transmitter transporters increasing their integrative capability (Borroto-Escuela et al. 2012, 2013b; Flajolet et al. 2008; Lee and Liu 2004; Liu et al. 2000).

Homo and heteroreceptor receptor complexes with allosteric receptor–receptor interactions give a new dimension to molecular neuroscience and brain integration and represents a new biological principle to integrate biological signals in all tissues. The structural determinants that decide if a receptor pair forms a heteromer or not exist in the receptor interface that mediates the allosteric receptor–receptor interaction (Woods et al. 2005). A substantial amount of work has been devoted to identify the key residues involved, including the development of a model of the A2AR–D2R heterodimer (Borroto-Escuela et al. 2018b). It is accepted that the transmembrane helices in class A, B and C dimers play a significant role (http://www.gpcr-hetnet.com) (Borroto-Escuela et al. 2014a). Interfaces usually possess complementary pockets in the core of the interface. The conserved amino acids that mediate the binding between the two interfaces are called hot spots and may be isolated from the surrounding solvent by adjacent amino acids (Bogan and Thorn 1998). Based on bioinformatic methods and original software we introduced the protriplet puzzle theory, stating that parts of triplet amino acid homologies can form hot spots and importantly participate in receptor–receptor interactions in receptor heteromers and other types of protein–protein interactions (Borroto-Escuela et al. 2018c; Tarakanov and Fuxe 2010; Tarakanov et al. 2011, 2012).

**Fig. 1:**
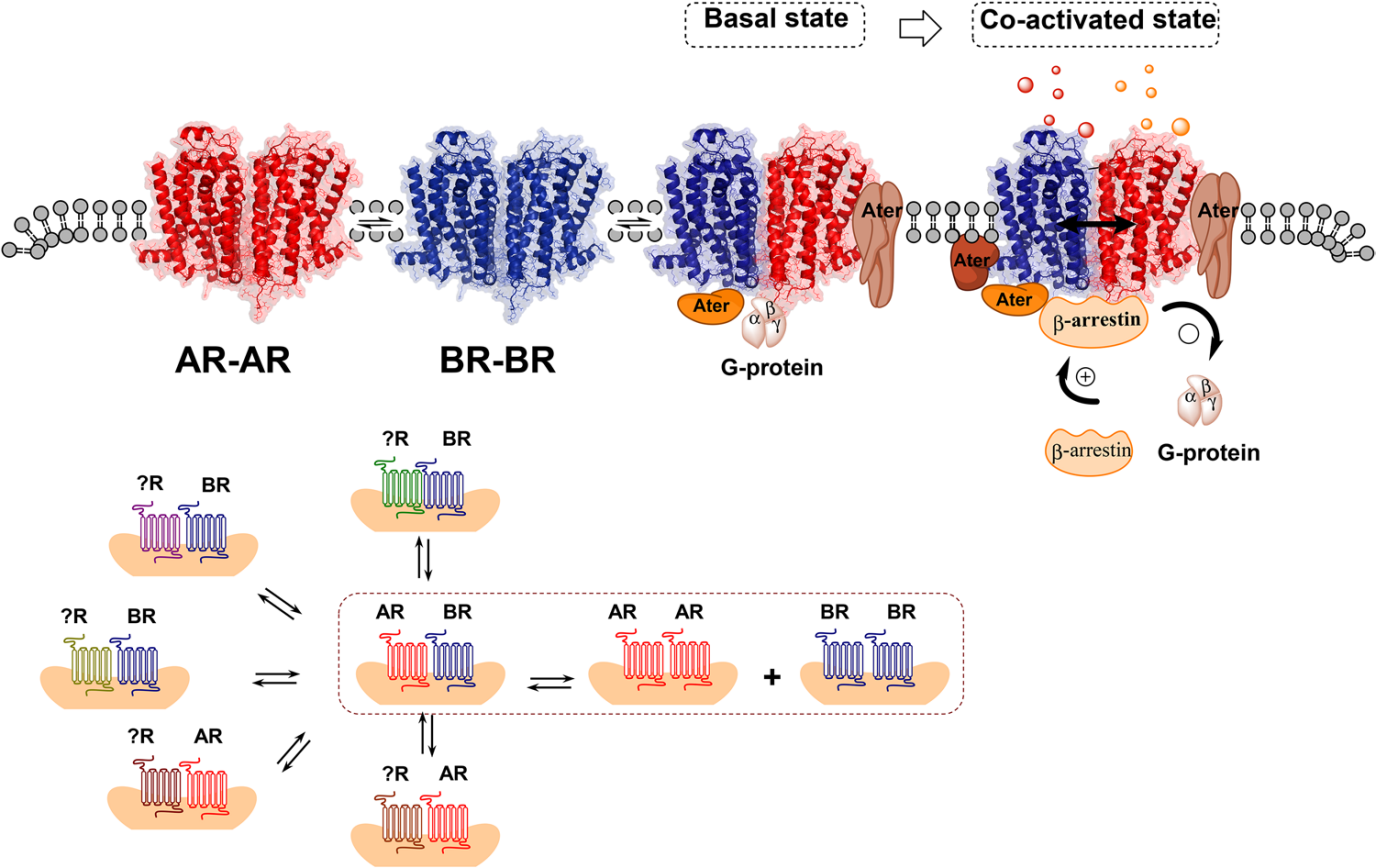
(Upper panel). Illustration of the fundamentals of the allosteric receptor–receptor interactions and the role of the receptor interface. A receptor heterodimer A–B is formed when certain amino acids in the two surfaces of the receptor interface of the heterodimer can bind to each other and form hot spots of considerable strength. Each heterodimer develops its own unique allosteric receptor–receptor interactions that must pass over the receptor interface, which can be located in transmembrane, intracellular and/or extracellular regions of the receptor. Upon, e.g., agonist activation of one receptor protomer the allosteric wave induced will pass over the receptor interface and induce either facilitatory or antagonistic allosteric changes in the function of the other receptor protomer. It can also involve a switch of, e.g, signaling over Gi/o to signaling via another G protein or beta-arrestin (bottom panel). A heterodimer A–B can also be in balance with other receptor complexes of different types (XA, XB) in a synaptic plasma membrane region or an extrasynaptic plasma membrane region. A disturbance of the balance among the receptor complexes can lead to dysfunction in transmission and to mental and neurological disorders

The signaling consequences of the association of two or several receptors leading to the formation of heteroreceptor complexes can be marked, dynamic and take place via the allosteric receptor–receptor interactions including the receptor–protein interactions, especially the adaptor proteins (Fig. 1). As a result, the GPCR protomers can switch their signaling, e.g., from Gi/o to Gq or to other signaling proteins like beta-arrestin (Fig. 1). The allosteric interaction is also reciprocal and GPCR protomer can also enhance or reduce the function of partner receptor. In complexes with receptor tyrosine kinases (RTK) it can allosterically modulate the trophic function of the partner RTK and in a complex with an ionotropic receptor it can modulate flow of ions through its ion channels. The allosteric receptor–receptor interactions are usually influenced in a dynamic way by activation of their respective ligands, especially upon coactivation of the two receptor protomers (Borroto-Escuela and Fuxe 2017; Fuxe et al. 2014c, e) (Fig. 1).

As to the biological logic of the homo-and heteroreceptor complexes, we proposed that the molecular basis of learning and memory can be represented by the reorganization of the homo- and heteroreceptor complexes in the postjunctional membrane of synapses (Borroto-Escuela et al. 2015a; Fuxe et al. 2014b). Thus, there is a need for integration of signals already at the plasma membrane. Changes in the prejunctional receptor complexes take place to facilitate the pattern of transmitter release to be learned. Long-term memory may be created by the transformation of parts of the heteroreceptor complexes into soluble molecules that can bind to transcription factors and modulate their DNA actions. This may lead to the formation of specific adapter proteins which by binding to the receptor complexes and scaffolding/cytoskeletal proteins can consolidate the homo–heteroreceptor complexes into long-lived complexes with conserved allosteric receptor–receptor interactions.

The formation of homo-and heteroreceptor complexes in a synaptic or extrasynaptic area of the plasma membrane is governed by several factors and especially by the density of the participating receptor protomers (Fuxe and Borroto-Escuela 2016). Another factor is the affinity of one receptor protomer for another protomer, which is related to the number of hot spots that can develop in the receptor interface (Borroto-Escuela et al. 2018b). The presence or absence of adapter proteins in the heteroreceptor complex can be a significant factor for determining the affinity that develops between two or more receptor protomers (Borroto-Escuela et al. 2018b). With an increase in the binding affinity between the two receptor protomers an increase in the number of receptor complexes can develop. Receptor agonists can modulate receptor complexes through conformational changes as determined in BRET experiments (Audet et al. 2008; Borroto-Escuela et al. 2013a, b) (Audet et al. 2008; Borroto-Escuela et al. 2013a).

There usually is a competition between different receptor complexes for the same receptor protomer since they are in balance with each other (Borroto-Escuela et al. 2015b, 2016, 2017a; Fuxe et al. 2014c, d). This is the case of a heteromer and its corresponding homomers and different heteromers sharing one or two receptor protomers (Borroto-Escuela et al. 2016, 2018d).

There in no rule for the allosteric receptor–receptor interactions in heteroreceptor complexes but are unique for each receptor heteromer. Either antagonistic or facilitatory receptor–receptor interactions develop upon agonist coactivation of the receptor protomers (Fuxe and Borroto-Escuela 2018; Fuxe et al. 2009a, b). They also markedly change when a receptor complex moves from a dimer to a trimer, e.g., GalR1–5-HT1A and GalR1–GalR2 (Borroto-Escuela et al. 2010b, 2014b) to a GalR1–GalR2–5-HT1A complex (Millon et al. 2016) and when the amount of adapter protein increases in the complex like cocaine recruitment of Sigma 1R into the A2A–D2 heteroreceptor complex (Borroto-Escuela et al. 2018d, e). The allosteric receptor–receptor interactions give diversity, bias and specificity to the signaling of each heteroreceptor complex.

A key question for the current work on A2AR–D2R heteroreceptor complexes is how to distinguish between the inhibitory physical A2AR–D2R interactions based on allosteric receptor–receptor interactions from the one based on downstream signaling mediated by G proteins. This question applies to all heteroreceptor complexes with inhibitory allosteric receptor–receptor interactions. The answer is that you should determine the receptor interface for the receptor complex of interest, in this case A2AR-D2R heteroreceptor complex. This was recently done and the A2AR TM5 peptide was found to have a key role in the A2AR–D2R interface (Borroto-Escuela et al. 2018e). Upon microinjection of this peptide into the ventral striatum it was found to block the A2AR agonist induced inhibition of cocaine self-administration and restore it to control values linked to a marked disappearance of the A2AR–D2R heteroreceptor complexes in the ventral striatum (Borroto-Escuela et al. 2018d, e).

## Adenosine receptors

Adenosine is a significant neuromodulator in the CNS that originates from the ATP located intracellularly and extracellularly in the neuronal and glial cells. Adenosine located intracellularly can reach the extracellular space via transporters present in the plasma membrane. Adenosine is also generated from ATP which upon exocytosis from neurons and glial cells is rapidly converted to adenosine by 5′ectonucleotidases, which are in abundance in striatum. It uses volume transmission for communication taking place in the extracellular space of the brain through diffusion and flow (Fuxe et al. 2010a). Adenosine receptors play a crucial role in mediating adenosine communication. There are four different subtypes namely A1R, A2AR, A2BR and A3R (Fredholm et al. 2011).

The A2AR is coupled to Gs/olf proteins and increases the adenylate cyclase (AC)–PKA–CREB pathway. It is found in high densities in the dorsal striato-pallidal GABA pathway. The A2AR enhances the activity of these neurons mainly through allosteric inhibition of the Gi/o mediated D2R protomer signaling in an A2AR–D2R heteroreceptor complex located in the soma-dendritic level of these GABA neurons (Fuxe et al. 2007b, 2010b). This activation of the dorsal striato-pallidal GABA pathway by A2AR agonists leads to motor inhibition (Fuxe et al. 2005). In contrast to the A2AR, the A2ABR has a low affinity (micromolar range) for adenosine and it is coupled to Gs/q involving PLC activation with increases in intracellular Ca^2+^ levels (Fredholm and Altiok 1994; Fredholm et al. 2011).

The A1R is instead a Gi/o coupled receptor and its activation leads to inhibition of AC and calcium channels while the potassium channels are activated (Gomes et al. 2011). The A1R exists all over the CNS in nerve cells and in glial cells and inhibits transmission and hyperpolarizes nerve cells. Of high interest for Parkinson’s disease (PD) is the location of the A1R in the direct GABA pathway from the dorsal striatum to the nigral (reticulata)/entopeduncular regions which initiates movements (Fuxe et al. 2008a). Finally, the A3R is coupled to the Gi/o and also inhibits AC signaling. However, it can also signal via PLC (Fredholm et al. 2011).

In view of the gene contribution to PD it is of high interest that a possible causative mutation can exist in the gene encoding the A1R. It is present in the PARK16 locus (Jaberi et al. 2016). Thus, a dysfunction of the A1R can contribute to the development of PD and a mutated A1R also appears to increase the vulnerability to PD. Understanding the consequences of A1R dysfunction in the direct pathway initiating movements (Surmeier et al. 2014) therefore, becomes of special interest.

In this review, we will give an update on the existence and function of adenosine isoreceptor and heteroreceptor complexes in the brain both in terms of transmission and of neurodegeneration.

## Adenosine isoreceptor complexes

By means of in situ proximity ligation assay (in situ PLA) it was possible to demonstrate the existence of A1R–A2AR, A2AR–A2AR, A2AR–A2BR, A2AR–A3R isoreceptor complexes, especially in the hippocampal formation (Borroto-Escuela et al. 2014a, 2016). With BRET it was also possible to demonstrate these four types of adenosine isomers in HEK293 cells (Cristovao-Ferreira et al. 2011, 2013). Previously with BRET A2AR–A2AR homodimers had been found in cellular models (Canals et al. 2004).

### A1R–A2AR heteroreceptor complexes

In 2006, A1R–A2AR isoreceptor complexes were identified in striatal glutamate neurotransmission at the presynaptic level (Ciruela et al. 2006). A tetrameric model of the A21R–A2AR isoreceptor complexes was proposed (Franco et al. 2014; Navarro et al. 2014). This model builds on indications that one A2AR homodimer and one A1R homodimer can come together via transmembrane V. One Gi/o protein can bind to the two receptor protomers of the A1R homodimer and one Gs protein to the two protomers of the A2AR homodimer. They are both necessary to obtain Gi protein mediated A1R signaling or Gs protein mediated A2AR signaling in this tetrameric A1R–A2AR isoreceptor complex.

It was demonstrated that high extracellular levels of adenosine could activate the A2AR protomer in this complex producing an antagonistic allosteric receptor–receptor interaction inhibiting A1R protomer signaling. The complex is located on the striatal glutamate nerve terminals and the reduction of the inhibitory A1R protomer signaling increases glutamate release (Franco 2009). It is important to determine if the A1R–A2AR isoreceptor complexes are located on all the incoming cortico-striatal and thalamo-striatal glutamate projections or only on some of them to understand their role in the basal ganglia circuits (Surmeier et al. 2014). At the moment, there is no clearcut evidence for a putative selective expression of A1R–A2AR isoreceptor complexes on the glutamate terminals of the striato-pallidal GABA nurons.

It should be considered that A2AR agonists produce inhibition of locomotion via both presynaptic (Shen et al. 2008) (Shen et al. 2008) and postsynaptic mechanisms (Fuxe et al. 2007b). An increase of striatal glutamate release was observed, especially after removal of the dopamine nerve terminals in models of PD (Ferraro et al. 2012; Fuxe et al. 2008a, c; Tanganelli et al. 2004). There is, therefore, support for the view that A2AR–D2R heteroreceptor complexes exist not only on the striato-pallidal GABA neurons (Fuxe et al. 2005) but also on the cortico-striatal glutamate nerve terminals, where the A2AR protomer inhibits the D2R protomer-induced inhibition of glutamate release. On these glutamate terminals, there may exist A1R–A2AR and A2AR–D2R heteroreceptor complexes in balance with an A1R–A2AR–D2R trimeric heteroreceptor complex.

It is proposed that the A1R–A2AR isoreceptor complexes with reciprocal antagonistic receptor–receptor interactions, leading to enhanced glutamate release upon A2AR activation, may possibly exist on glutamate projections regulating the striato-pallidal GABA neurons mediating motor inhibition. The reason is that A2AR agonists inhibit movements which should not be the case if the A1R–A2AR complex is also located on the glutamate terminals regulating the direct pathway which initiates movements (Surmeier et al. 2014). In this case, the A2AR induced release of glutamate should counteract inhibition of movements. Future research will determine if this proposal is of value. If involved, it would mean that A2AR protomer when activated by high extracellular levels of adenosine in the glutamate synapses on the indirect pathway could more effectively increase glutamate release by inhibition of not only the D2R protomer but also the A1R protomer. This molecular integration at the presynaptic level will add to the enhancement of firing through the antagonistic A2AR–D2R interaction in the postjunctional receptor complex of the striato-pallidal GABA neurons increasing motor inhibition (see below).

### A2AR–A2BR heteroreceptor complexes

The A2BR is expressed in many brain regions in low–high densities and their distribution overlaps with the A2ARs in the brain (Fredholm 1995; Fredholm et al. 2011). Both of these receptors are overexpressed in pathological conditions, e.g., neuroinflammation.

It is of high significance that the A2ARs and A2BRs were demonstrated to heteromerize and form heteroreceptor complexes (Borroto-Escuela et al. 2015b, 2016; Hinz et al. 2017). BRET, bimolecular fluorescence complementation (BiFC) and in situ PLA were used to demonstrate their existence in cotransfected CHO cells (Hinz et al. 2017). The major finding of this work was the ability of A2BR protomer to block the recognition and signaling of the A2AR protomer (Hinz et al. 2018). Thus, the A2AR agonist lost its high affinity binding to the A2AR protomer upon coexpression of the A2BR. As a result, the A2AR signaling was counteracted as demonstrated from the failure of the A2AR agonist to increase cAMP accumulation.

It should be noticed that the presence of the A2BR protein itself was sufficient to inhibit the function of the A2AR. The activation of theA2BR with an A2BR agonist was not needed. Thus, the two receptor proteins must have a high affinity for each other. With increased expression of the A2BR a complete block of the A2AR could be obtained.

Of particular relevance is their demonstration in native tissue like the brain using in situ PLA (Borroto-Escuela et al. 2015b; Fuxe and Borroto-Escuela 2018). So far, A2AR–A2BR specific clusters were found in moderate–high densities in the dorsal hippocampus, mainly involving the pyramidal cell layer. The size of the PLA-positive clusters ranged between 0.5 and 2 µm. Their distribution in the brain is presently being studied.

These findings are of special interest, since A2AR is involved in mediating neurodegeneration (Borroto-Escuela et al. 2018a; Ferreira et al. 2017; Hu et al. 2016; Laurent et al. 2016). Increased expression of A2BR in the brain can, therefore, be a novel strategy for counteracting A2AR-mediated neurodegeneration, provided that they can be formed in the A2AR positive neurons and/or reach such neurons via extracellular vesicle-mediated volume transmission. This can involve the internalization of, e.g., exosomes, transporting A2BR, into the A2AR expressing neurons (Agnati and Fuxe 2014; Borroto-Escuela et al. 2015a). The origin can be both be neuronal and glial (Borroto-Escuela et al. 2015a). It is presently unknown if the A2BR is primarily located on neuronal or glial cells.

## Adenosine heteroreceptor complexes

### A2AR–D2R heteroreceptor complexes

By means of in situ PLA these receptor complexes were identified in high densities mainly in the dorsal striatum and in the ventral striatum. They were observed as red clusters with a size range of 0.5–2 µm located in the neuropil outside the nuclei (Borroto-Escuela et al. 2013c; Fuxe and Borroto-Escuela 2018; Trifilieff et al. 2011). The results indicate that they are located mainly in the dorsal and ventral striato-pallidal GABA pathways but also in cholinergic striatal interneurons and in cortico-striatal glutamate terminals (Borroto-Escuela et al. 2016, 2017b, 2018f; Feltmann et al. 2018; Fuxe et al. 2007b, c; Shen et al. 2008). They are constitutive and were also demonstrated in cellular models with BRET/FRET techniques (Borroto-Escuela et al. 2010a, c; Canals et al. 2003; Hillion et al. 2002).

Their antagonistic allosteric receptor–receptor interactions led to a strong reduction of the affinity of the high affinity binding of the D2R protomer and to a significant inhibition of the Gi/o mediated D2R protomer signaling (Azdad et al. 2009; Borroto-Escuela et al. 2010a, c; Fuxe et al. 2003, 2007b). The signaling of the D2R protomer was instead dominated by beta-arrestin mediated signaling (Borroto-Escuela et al. 2011, 2013b). It was beautifully demonstrated by Surmeier and colleagues that D2R mediated long-term depression was antagonized by an A2AR agonist and a return of long-term potentiation was observed (Shen et al. 2008). The results indicate the possibility that the A2AR–D2R heterorecepor complexes can play a significant role in neuroplasticity. It should be noted that interactions exist between calmodulin, a calcium binding protein, A2AR and D2R (Navarro et al. 2009). Calmodulin bound to this heteroreceptor complex at the proximal C-terminus of the A2AR. Calcium produced changes in the structure of the trimeric complex that correlated with the modulation of MAPK activity in the A2AR as well as in the D2R.

### The hemiparkinson model in the rat

It is known from the work of Urban Ungerstedt that the striatal D2R on the lesioned side with loss of striatal DA nerve terminal networks develop D2R supersensitivity (Fuxe et al. 2015; Kostrzewa et al. 2018). In spite of supersensitivity development, the increase in antagonistic allosteric A2AR–D2R interactions were observed in the dorsal striatal membranes on the lesioned side (Ferre and Fuxe 1992; Fuxe et al. 1992, 1993, 2015; Navarro et al. 2016). These results strongly indicated that A2AR antagonists will represent novel antiparkinson drugs as shown by the marked enhancement of contralateral turning behavior induced by dopamine receptor agonists (Stromberg et al. 2000). It is also known that subthreshold doses of D2like receptor agonists can produce locomotion following pretreatment with A2AR antagonists (Tanganelli et al. 2004). The A2AR–D2R complexes are mainly located in the dorsal striato-pallidal GABA neurons mediating motor inhibition. The inhibitory actions of Gi/o mediated D2R protomer signaling removes this brake but A2AR protomer activation will restore it.

One mechanism for the enhanced A2AR–D2R interactions in models of PD can be an increase in the density of the A2AR–D2R heteroreceptor due to an increased affinity for each other related to the conformational change in the supersensitive D2R. The D2R can be recruited from D2R homodimers leading to a small reduction in the density of the D2R homoreceptor complexes. Furthermore, the supersensitivity developed in the D2R protomer (Kostrzewa et al. 2018) and its conformational change can also contribute to the enhanced A2AR–D2R interactions.

Early on in treatment of PD with levodopa and D2R agonists can overcome the basal A2AR protomer inhibition of the D2R protomer. One reason can be that the density of D2R homoreceptor complexes is still higher than the density of the A2AR–D2R heteroreceptor complexes and the therapeutic actions can develop. However, as treatment continues complications develop with development of dyskinesias and wearing off of the therapeutic effects. The mechanism for these actions can be an increased internalization of the D2R protomers in A2AR–D2R heteroreceptor complexes and D2R homoreceptor complexes leading to an increased dominance of A2AR homoreceptor complexes. In addition, there are indications that increased phospho-CREB can be formed upon chronic activation of the D2R and D2R protomers through increased activation of CRE in the A2AR promotor region. This leads to increased expression of A2AR (Antonelli et al. 2006). As a result, a dominance of A2AR homoreceptor complexes and to some degree of through A2AR–D2R heteroreceptor complexes develop versus the D2R monomers and D2R homoreceptor complexes and a wearing off of the therapeutic effects take place. The motor inhibition increases through overactivity in the striato-pallidal GABA neurons. These mechanisms can also help explain the dyskinesias that develop upon chronic treatment with the dopaminergic drugs. Movements initiated via the D1R in the direct pathway to the internal segment of the globus pallidus and zona reticulata of the substantia nigra can no longer lead to appropriate movements, since the motor brake via the striato-apllidal GABA pathway can no longer match the movements initiated (Fuxe et al. 2015). We are aware of the modest effects observed with A2AR antagonists in PD so far (Hauser 2011). Thus, there are a number of other molecular and receptor mechanisms that also are involved and play a significant role (Shadfar et al. 2018; Surmeier et al. 2017a, b).

### The MitoPark model

The midbrain dopamine nerve cells develop a progressive degeneration in the MitoPark model as a result of a cell-type specific mitochondrial dysfunction leading to progressive parkinsonism (Ekstrand et al. 2007). The mice in this model can be described as conditional knockout mice with disruption of the gene for the mitochondrial transcription factor of the dopamine nerve cells. The MitoPark animals mirror the slow progression of key symptoms in PD (Galter et al. 2010).

In this model we used the specific A2AR antagonist MSX-3 (Marcellino et al. 2010; Sauer et al. 2000). We studied the locomotion in the MitoPark mice following daily treatment for 8 weeks with MSX-3 with or without levodopa in low or high doses of levodopa (Marcellino et al. 2010). Chronic treatment with MSX-3 alone was found to block the progressive reduction of spontaneous locomotion (measured 24 h after the last injection) found in saline controls and levodopa treated mice. The ability of MSX-3 but not levodopa to maintain locomotion was clearly observed and significant, but the gradual disappearance of striatal dopamine levels was not counteracted. Thus, neuroprotective effects of the A2AR antagonist did not seem to be involved. Our hypothesis is that the antiparkinson effects of the early A2AR antagonist treatment was related to a blockade of a reorganization of the A2AR homoreceptor complexes A2AR–D2R heteroreceptor complexes and their balance with the D2R homoreceptor complexes that may develop during the progressive degeneration of the nigral dopamine neurons in this model (Borroto-Escuela et al. 2017a; Fuxe et al. 2015). The blockade of the A2AR may reduce the affinity of the A2AR protomer and the D2R protomer for each other and instead the D2R protomer will prefer to form D2R homoreceptor complexes. These D2R homoreceptor complexes lacking the A2AR brake will be in dominance and explains the maintenance of locomotion 24 h after the A2AR antagonist treatment.

Instead, the daily treatment with levodopa can lead to increased internalization of D2R and their activation by levodopa can lead to increased expression of A2AR participating in homo and A2AR–D2R heterorecepor complexes (Antonelli et al. 2006; Fuxe and Borroto-Escuela 2016; Fuxe et al. 2015). Thus, in this case the A2AR–D2R and A2AR homoreceptor complexes may dominate 24 h after the levodopa treatment. The hypothesis builds on the dynamic equilibrium between D2R and A2AR homoreceptor complexes and A2AR–D2R heteroreceptor complexes that can explain the pharmacological findings on locomotion in the MitoPark model.

### A2AR–D2R–mGluR5 heteroreceptor complexes

These heteroreceptor complexes are also located mainly extrasynaptically to glutamate synapses on the dorsal striato-pallidal GABA neurons and regulate their GABA transmission (Beggiato et al. 2016; Cabello et al. 2009; Fuxe et al. 2003, 2010b). They are in equilibrium, especially with A2AR–D2R heteroreceptor complexes and their corresponding A2AR, D2R and mGluR5 homoreceptor complexes. They are of high interest since A2AR and mGluR5 synergize to inhibit D2R protomer recognition and signaling over the MAPK and pCREB intracellular pathways via antagonistic allosteric receptor–receptor interactions (Ferre et al. 2002; Fuxe et al. 2003; Popoli et al. 2001). Behavioral results show in line with these results that combined treatment with mGluR5 and A2AR antagonists are particularly effective in producing antiparkinson actions (Schwarzschild et al. 2006).

In Parkinson’s disease, the dopamine nerve terminals degenerate and the D2R on the glutamate nerve terminals can no longer appropriately inhibit glutamate release. As a result, the synaptic release of glutamate and ATP becomes enhanced with ATP broken down to adenosine which can activate the mGluR5 and A2AR protomers in the postjunctional A2AR–D2R–mGluR5 complex, respectively. Thus, an increased brake on the D2R signaling develops. As discussed above, it is also possible that the increased activation of A2AR monomers–homomers and mGluR5 homomers by increased extracellular levels of adenosine and glutamate can increase their affinity for D2R and for each other in the plasma membrane and thus enhanced formation of the A2AR–D2R, A2AR–mGluR5 and the trimeric receptor complexes above can take place. As a consequence the D2R homoreceptor complexes become reduced since many have been recruited to the heteroreceptor complexes above.

It seems possible that this reorganization can be reduced by A2AR antagonist and/or mGluR5 antagonist treatment reducing the recruitment of A2AR and mGluR5 to the D2R. The therapeutic effects of the levodopa and D2R agonists can become increased, the onset of their treatment delayed and the wearing off of their anti-parkinson actions reduced. Our hypothesis is that the wearing off of the antiparkinson actions may be a progressive increase over time of the brake on the inhibitory D2R protomer signaling on the striato-pallidal GABA neurons increasing motor inhibition. The mechanism can involve increased expression of the A2AR and mGluR5, increased internalization of activated D2R with reduced recycling via endosomes to the plasma membrane leading to a dominance of A2AR–D2R and A2AR–D2R–mGlu5R heteroreceptor complexes.

It is known that both D2R agonists and D1R agonists can produce dyskinesias (Luquin et al. 1992; Rascol et al. 2001). It seems likely that the balance in activity between the D2R regulated striato-pallidal GABA neurons (indirect pathway) inhibiting movements and the D1R regulated striato-internal pallidum/nigral GABA pathway (direct pathway) is of importance to stop dyskinesia development. A disbalance may lead to dyskinesias. It seems that a complete removal of the brake on D2R signaling and/or a reorganization of the A2AR and D2R homo and heteroreceptor complexes that favor marked exaggerated D2R protomer signaling with loss of motor inhibition leads to dyskinesias. In this case, the movement initiated by the direct pathway cannot be properly accompanied by a correct inhibition from the indirect pathway. The balance between the indirect D2R regulated and the D1R regulated direct pathway appears fundamental to avoid dyskinesia development. Such a view may help to understand the anti-dyskinetic effects of a number of NMDA receptor antagonists including amantadine in treatment of levodopa-induced dyskinesias (Brigham et al. 2018; Calon and Di Paolo 2002). The NMDA receptors drive both the direct and indirect pathways and may have a differential pharmacology-different NMDAR complexes and subunit structure in the two pathways. As a result the balance may be restored by several NMDAR antagonists and a reduction of the dyskinesias can take place.

Based on the studies on, e.g., A2AR–D2R and A2AR–D2R–mGluR5 heteroreceptor complexes new strategies for treatment of PD can be developed. Brain penetrant heterobivalent compounds with A2AR and mGluR5 antagonist pharmacophors will have the potential to target the A2AR–D2R–mGluR5 complexes. Instead heterobivalent compounds with A2AR antagonist and D2R agonist pharmacophors could target both the A2AR–D2R and A2AR–D2R–mGluR5 heteroreceptor complexes. Such compounds should help remove the brake on the D2R signaling in PD and have potential advantages compared with D2R agonists in view of possible increases in target specificity.

### A2AR–D3R and A2AR–D4R heteroreceptor complexes

Indications that A2AR can heteromerize also with dopamine D3Rs were obtained in cellular models in 2005 using FRET (Torvinen et al. 2005). Antagonistic A2AR–D3R interactions were also demonstrated leading to reduction of the affinity of the high affinity D3R agonist binding site. The receptor interface involved also for this receptor heteromer electrostatic forces between positively charged arginines (D3R intracellular loop 3) and negatively charged aspartates (A2AR C-terminus) (Fuxe et al. 2005). The D3Rs in brain are mainly found in the ventral striatum unlike the D2R which is located in high densities in both ventral and dorsal striatum. These results strengthen the view that A2AR agonists should counteract psychosis development in PD patients upon dopaminergic treatment by targeting A2AR–D2R and A2AR–D3R heteroreceptor complexes in the ventral striatum (Fuxe et al. 2010b).

**Fig. 2:**
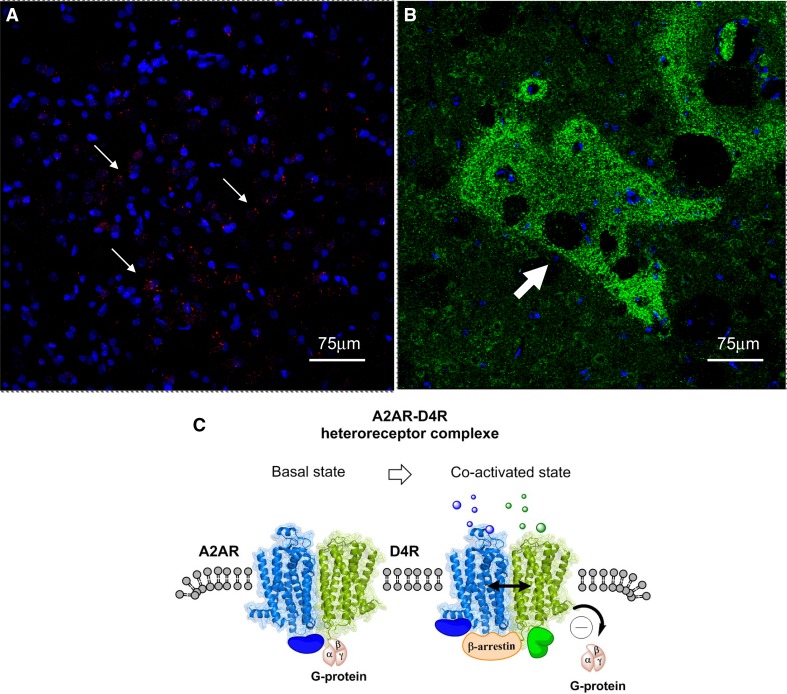
**a** The red PLA positive clusters of A2AR–D4R heteroreceptor complexes are enriched in a distinct region of the sampled field from the dorsal striatum. White arrows point to some of the red PLA positive clusters. Nuclei have a blue color. **b** Green D4R immunoreactivity is shown as densely packed green dots forming islands (see white arrow) (striosomes) in the dorsal striatum. **c** A2AR–D4R heterodimers in the plasma membrane in the basal state and in the co-activated state. Upon coactivation of the A2AR protomer (Blue) and the D4R protomer (green) the D4R induced increase in Gi/o protein signaling is probably inhibited by the antagonistic allosteric A2AR–D4R interaction. At the same time beta-arrestin can be recruited to the A2AR–D4R heterodimer and become part of the A2AR–D4R signaling. The blue and green solid spheres represent different types of adapter proteins bound to the heterodimer and upon coactivation a new adapter protein (in green) can be recruited to the heterodimer

It was early on indicated that also A2AR and dopamine D4R can form heteroreceptor complexes based on their interface which involved electrostatic interactions as outlined above for the A2AR–D2R (Borroto-Escuela et al. 2010a) and A2AR–D3R (Fuxe et al. 2005). It was demonstrated by Rivera et al. in 2002 that the D4R was enriched in the striosomes but also found in the matrix of the dorsal striatum (Rivera et al. 2002). It is, therefore, of high interest that Borroto-Escuela et al. demonstrated A2AR–D4R heteroreceptor complexes in the dorsal striatum, especially in the striosomes using the in situ PLA method (Fig. 2) (Borroto-Escuela et al. 2016; Fuxe and Borroto-Escuela 2018).

The striosomes (Graybiel and Ragsdale 1978) using GABA as transmitter can project directly to the nigral DA neurons and inhibit their activity (Crittenden and Graybiel 2011; Crittenden et al. 2016; Fujiyama et al. 2011; Shumilov et al. 2018; Watabe-Uchida et al. 2012). It is, therefore, of high interest that A2AR–D4R heteroreceptor complexes can in part be located to the striosomal GABA neurons. It seems likely that also in this receptor complex an antagonistic allosteric receptor–receptor interaction can develop reducing the Gi/o mediated inhibitory D4R signaling. This may result in an enhanced activity of the striosomal GABA neurons directly projecting to nigral dopamine cells. As a result, a reduced activity with reduced dopamine turnover should develop in the dopamine nerve cells projecting to the striosomes.

It is of substantial interest that an ergolene derivative MPME, being in part a partial dopamine D1R agonist, can preferentially reduce dopamine turnover in the striosomes versus the matrix and the dotted type of ventral striatal dopamine terminals (Fuxe et al. 1978; Ogren 1985). These results in the striosomes can be explained by a special D1R pharmacology that preferentially activates the D1Rs of the striosomes. The striosomal GABA projection to the nigral dopamine cells increases its activity and the nigral dopamine projection to the striosomes becomes inhibited. As a result, the striosomal dopamine turnover is preferentially reduced. It is of high interest that a selective ablation of the striosomes leads to an increase of dopamine transmission in the striosomes and a reduction of dopamine transmission in the striatal matrix (Shumilov et al. 2018) which is in line with our previous findings. Thus, there seem to exist nigral dopamine neurons that preferentially project to the striosomes or to the striatal matrix. The inhibition of matrix dopamine transmission upon striosome ablation may be related to the absence of GABA collaterals from the striosome projection neurons reaching GABA interneurons in the zona reticulata of the substantia nigra (Shumilov et al. 2018). As a result, the GABA interneurons are disinhibited and can inhibit dopamine transmission in the nigral dopamine neurons projecting to the striatal matrix (Shumilov et al. 2018).

It should be noticed that also D4R–MOR heterorecepor complexes appear to exist in the striosomes (Rivera et al. 2017). Facilitatory allosteric D4R–MOR interactions were observed in striosome nerve cells enhancing the affinity of MOR, which should enhance inhibition by MOR of the striosome GABA projection neurons. These results serve to illustrate that multiple heteroreceptor complexes perform molecular integration also exist in the striosome projection neurons involving in this case A2AR–D4R complexes (Borroto-Escuela et al. 2016; Fuxe and Borroto-Escuela 2018). The D4R seems to have taken over the role of the D2R in the striosome system and interacts both with A2AR protomers and MOR protomers.

### A1R–D1R heteroreceptor complexes

Antagonistic A1R–D1R receptor–receptor interactions were indicated to exist in the direct GABA pathway from the dorsal striatum to the entopeduncular/nigral regions in rodents using biochemical binding, pharmacological, microdialysis and behavioral studies (Franco et al. 2007; Fuxe et al. 1998, c, 2001, 2007a; Popoli et al. 1996; Rimondini et al. 1998). Coimmunoprecipitation in cellular models indicated the existence of A1R–D1R heteroreceptor complexes (Gines et al. 2000). With FRET/BRET techniques evidence for their existence could be obtained (Franco et al. 2007). Biochemical binding studies demonstrated that in striatal membranes the A1R agonist reduced the proportion of D1R in the high affinity state providing indications for antagonistic allosteric A1R–D1R interactions in the heteroreceptor complexes (Fuxe et al. 1998). Later on the distribution of the A1R–D1R heteroreceptor complexes in the rodent brain were mapped out with the proximity ligation assay (PLA) (Borroto-Escuela et al. 2016).

It was early on found that A1R agonists can reduce oral dyskinesias induced by levodopa in rabbits (Ferre et al. 1994). Observations that D1R agonists can disrupt the A1R–D1R heteroreceptor complexes in cells give a possible mechanism, especially since an A1R agonist blocked the D1R action (Gines et al. 2000). It seems possible that such events can develop also in the dorsal striatum in models of PD. The A1R agonist treatment may block the ability of levodopa to disrupt the A1R–D1R heteroreceptor complexes which may lead to anti-dyskinetic actions.

Such effects of levodopa producing a disappearance of the A1R–D1R heterorecepor complexes in the direct GABA pathway should lead to an increase in the number of D1R heteroreceptor complexes and overactivation of the D1R signaling with levodopa treatment. It is known that the D1R activation increases excitability and long-term potentiation at glutamate synapses on the direct pathway (Surmeier et al. 2014). Increased transcription factor activation develops over the D1R–AC–PKA–pCREB pathway. Therefore, multiple changes develop, e.g., with increased formation of GPCR interacting proteins modulating a number of heteroreceptor complexes and their signaling. In addition, D1R induced activation of DARPP-32 Thr34 produces protein phosphatase 1 (PP1) inhibition with increased phosphorylation in a number of receptor complexes and ion channels in glutamate synapses on the direct GABA pathway. Sensitization of the D1R will, therefore, have a major impact on the molecular integration of the glutamate synapses leading to an upstate of the direct pathway operating via GABA transmission to initiate movements. A stronger D1R mediated transmission has also been reported to take place in dyskinesia (Farre et al. 2015).

As discussed earlier a disbalance between the activities of the direct and indirect pathways can lead to dyskinesias. In this, case the balance may be disturbed by a pathological enhancement of the D1R signaling of the direct pathway produced by levodopa in the treatment of PD. As a result a pathological enhancement of the activity in the direct pathway initiating movements develops that cannot be appropriately matched by inhibition of movements through the D2R regulated indirect pathway.

### Putative A1R–D1R–D3R heteroreceptor complexes

Levodopa induced dyskinesias in a monkey model of PD was associated with an increased expression of the D3R in the striatum. Treatment with a partial D3R agonist led to an attenuation of the dyskinesia indicating a role for D3R-mediated dopamine transmission in dyskinesia (Bezard 2003). These findings became even more interesting when we demonstrated D1R–D3R heteroreceptor complexes with synergistic allosteric receptor–receptor in the striatum (Fiorentini et al. 2008; Marcellino et al. 2008). Thus, it was proposed that the levodopa induced dyskinesias can involve the expression of D3R in the direct pathway with formation of D1R–D3R heteroreceptor complexes in which the D3R protomer enhances the D1R protomer signaling and recognition.

It was also proposed that A1R–D1R–D3R heteroreceptor complexes may exist in equilibrium with the corresponding homoreceptor complexes and the A1R–D1R and D1R–D3R heteroreceptor complexes (Fuxe et al. 2008b). It remains to be demonstrated if they are formed and if so, the A1R agonist is still capable of bringing down the D1R protomer signaling in spite of its interaction with the D3R protomer. The question is also if A1R agonist treatment can increase both the A1R–D1R and A1R–D1R–D3R heteroreceptor complexes in the striatum, which can be part of its antidyskinetic actions.

## A2A receptors and their interactions with alpha-synuclein

It is known that A2AR can modulate alpha-synuclein aggregation and toxicity (Ferreira et al. 2017) and vice versa since alpha-synuclein can cause an increase of A2AR signaling. The A2AR homo and heteroreceptor complexes may participate in this process (Borroto-Escuela et al. 2018a). Cell death caused by alpha-synuclein could be blocked by A2AR antagonists as well as by genetic deletion of A2AR (Ferreira et al. 2017). This counteraction is probably related to the observation that A2AR agonists can increase the flow of calcium through the NMDAR channels (Rebola et al. 2008) as also found after alpha-synuclein (Diogenes et al. 2012). Such actions can cause excitotoxicity in glutamate synapses (Besancon et al. 2008) and the neurodegeneration is associated with deficits in cognition (Hu et al. 2016).

**Fig. 3:**
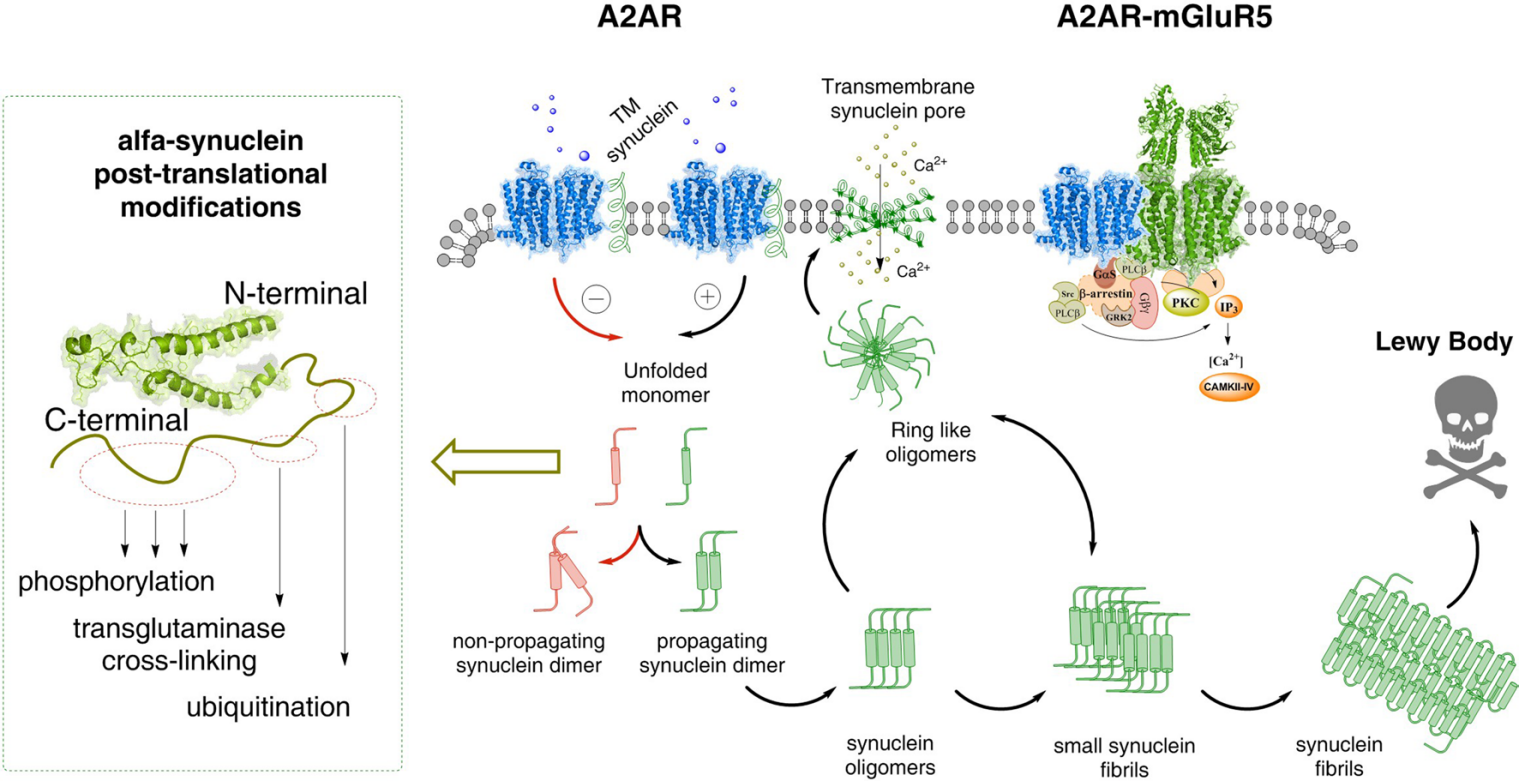
Possible molecular mechanism by which alpha-synuclein monomers/oligomers/synuclein fibrils can modulate the A2AR homo-heteroreceptor complexes and their balance in the plasma membrane. In the left part it is proposed that monomeric alpha-synuclein transmembrane (TM) peptides can become linked to the A2AR homoreceptor complex and modulate the A2AR function. Under the modulation of the monomeric alpha-synuclein peptides the A2AR antagonist may favor the formation of non-propagating alpha-synuclein dimers (pathway highlighted in red). Instead the A2A receptor agonist induced A2AR activation (pathway highlighted in green) may in the alpha-synuclein-A2AR complex produce signals that favor the propagation of alpha-synuclein dimers/oligomers into small and large synuclein aggregates that accumulate in Lewy bodies.In the far left several mechanisms are illustrated that can produce posttranslational modifications of alpha-synuclein. It involves changes in phosphorylation, transglutaminase cross-linking and ubiquitination and may play a role in the transformation of alpha-synuclein into propagating alpha-synuclein dimers. Ring-like synuclein oligomers can also be formed, which enter the plasma membrane and there produce beta sheet structures that associate and produce pores through which calcium ions may pass. In the A2AR–mGluR5 heteromer, shown as the coming together of two homodimers (A2AR homodimer in blue and mGluR5 homodimer in green), the signaling pathways are illustrated. Changes in the activity of protein kinases like PKA, PKC and calcium–calmodulin kinase II can have a role in the modulation of the synuclein aggregation process

It is unknown which forms of alpha-synuclein (monomers, dimers, higher order homomers) cause the increase of A2AR signaling in homo and heteroreceptor complexes in the plasma membrane of the striato-pallidal GABA neurons where the A2AR are mainly expressed. It can lead to increased formation of alpha-synuclein fibrils in the dendrites of these neurons, which are not vulnerable to Parkinson’s disease (Surmeier et al. 2017b). One possibility is that the monomers–dimers in the alpha helix conformation can bind to the A2AR and form an A2AR–alpha-synuclein complex. The direct receptor–protein interactions can enhance the A2AR signaling over the Gs/olf–AC–PKA intracellular pathway and phosphorylation of alpha-synuclein takes place (Oueslati 2016) (Fig. 3). However, there is an ongoing discussion whether the enhanced alpha-synuclein phosphorylation brings down or increases alpha-synuclein aggregation and the ability to produce degeneration. It may be that A2AR phosphorylates also an adapter protein which increases its binding to alpha-synuclein and alters its conformation. As a result, the fibrillation process of the alpha-synuclein homomers can become enhanced and increased neurodegeneration develops.

It may be that such events can also change the deubiquitination/ubiquitination of the C-terminal residues of the alpha-synuclein fibrils (Oueslati 2016) (Fig. 3). This may lead to reduced autophagy by diminished clearance of the alpha-synuclein fibrils over the lysosome degradation pathway (Decressac and Bjorklund 2013). Thus, the A2AR mediated production of the alpha-synuclein induced neurodegeneration may take place both by enhanced formation of alpha synuclein fibrils and by reducing their clearance.

Transglutaminase crosslinking in the C terminal residues of alpha-synuclein and truncation may play a role in modulating the A2AR induced events in the fibrillation process (Fig. 3). In cellular models, we will study the potential formation of A2AR–alpha-synuclein complexes with enhancement of A2AR signaling. The starting point will be the monomeric alpha-synuclein transmembrane peptide.

### Putative A2AR–D2R–NMDAR heteroreceptor complexes

It was suggested that A2AR activation can produce excitotoxicity via increasing NMDA receptor activity leading to increased influx of calcium over the NMDAR channels (Rebola et al. 2008). Such actions are also produced by alpha-synuclein (Diogenes et al. 2012). To understand the mechanism it should be considered that D2R–NMDAR heteroreceptor complexes exist (Liu et al. 2006). In this receptor complex, the D2R can interact with the NR2B subunit of the NMDAR and inhibit the NMDAR signaling. This heteroreceptor complex is located in the striato-pallidal GABA neurons mediating motor inhibition (Surmeier et al. 2014). In agreement, NR2B receptor antagonists were found to produce antiparkinsonian effects (Loschmann et al. 2004) probably by reducing the glutamate drive on the striato-pallidal GABA neurons causing reduction of motor inhibition mediated via the indirect pathway.

**Fig. 4:**
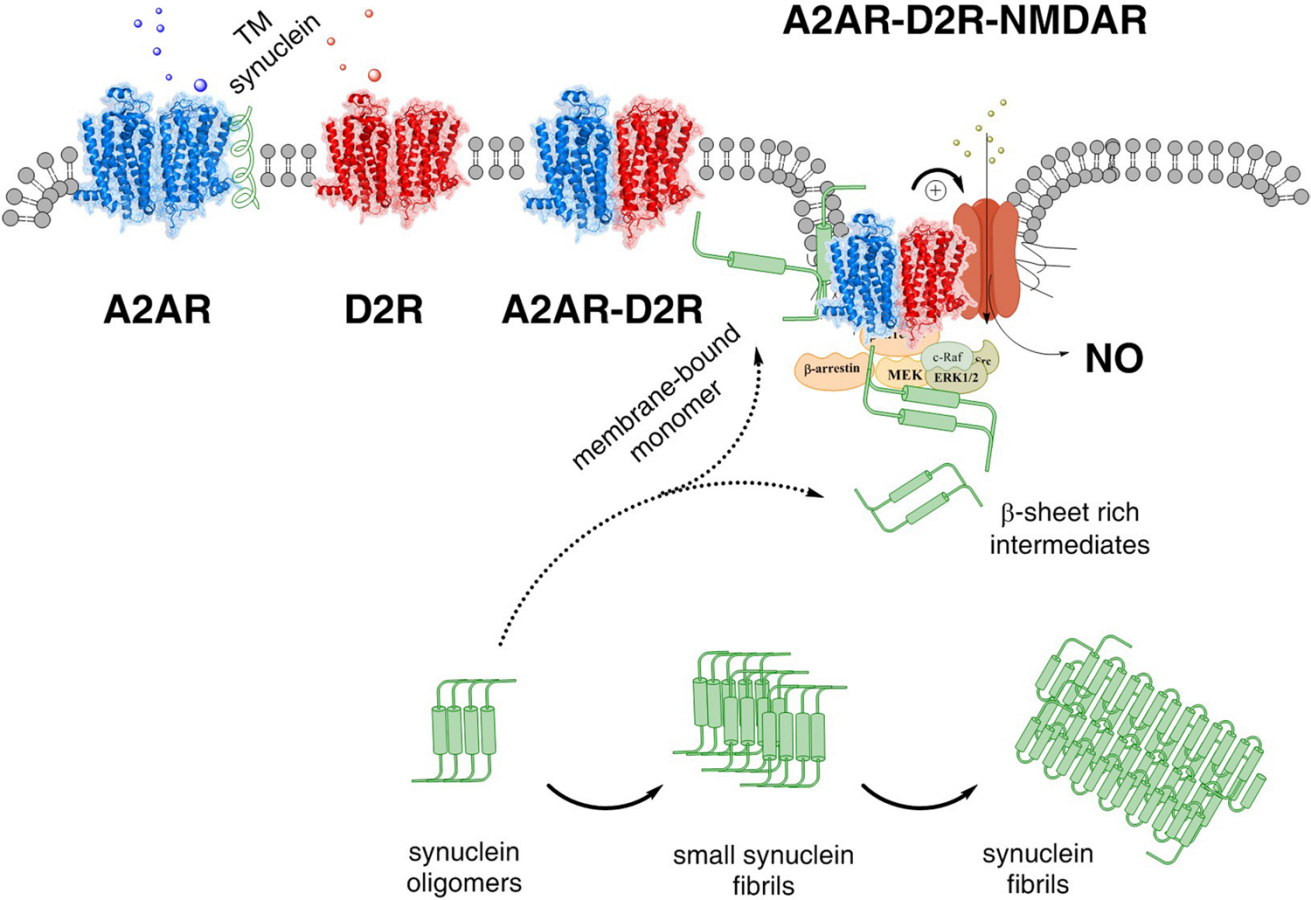
The A2AR–D2R–NMDAR complex and its balance with A2AR–A2AR and D2R–D2R homodimers and A2AR–D2R heterdimers are illustrated. The alpha-synuclein monomer (alpha conformation) may also bind to the A2AR protomer of the A2AR–D2R–NMDAR complex, enhancing A2AR protomer activation and its allosteric inhibition of the D2R signaling. As a result the allosteric inhibition by the D2R protomer of the NMDAR protomer is reduced and NMDAR function may become substantially enhanced with increased calcium influx through its ion channels (see + sign) leading to a coupling to nitric oxide (NO) production and toxicity. Beta sheet rich intermediates of alpha-synuclein peptides are proposed to bind to the intracellular loops and C terminal of the receptor protomers of this heteroreceptor trimeric complex and modulate their signaling. In addition, the signaling of the G proteins and beta-arrestin as well of other associated proteins like kinases in the signaling pathways may become modulated

It is, therefore, of substantial interest that A2AR–D2R heteroreceptor complexes also are located in these GABA neurons (Fig. 4). Both the A2AR–D2R and the D2R–NMDAR heteroreceptor complexes are colocated in the synaptic and extrasynaptic regions of the glutamate synapses on the striato-pallidal GABA neurons.

Based on these results it seems likely that there can exist an equilibrium not only between these heteroreceptor complexes and their corresponding homoreceptor complexes but also between A2AR–D2R and D2R–NMDAR complexes in the postjunctional regions of the glutamate synapses. In this equilibrium also putative A2AR–D2R–NMDAR can exist (Fig. 4).

The existence of such trimeric heteroreceptor complexes can give one molecular mechanism for how alpha-synuclein and A2AR activation can produce an increase in calcium influx over the NMDAR channels leading to enhanced activity in the striato-pallidal GABA neurons and other neurons possessing these trimeric complexes.

The hypothesis states that alpha-synuclein monomer–dimers are recruited to the plasma membrane where they form a complex with the A2AR enhancing its signaling. As a result, the allosteric brake on D2R signaling is increased with removal of the D2R brake on the NMDAR signaling. The NMDAR signaling is increased with enhanced calcium influx and increased activity develops in the striato-pallidal GABA neurons, which inhibit movements but are not vulnerable to neurodegeneration in Parkinson’s disease (Surmeier et al. 2017b).

**Fig. 5:**
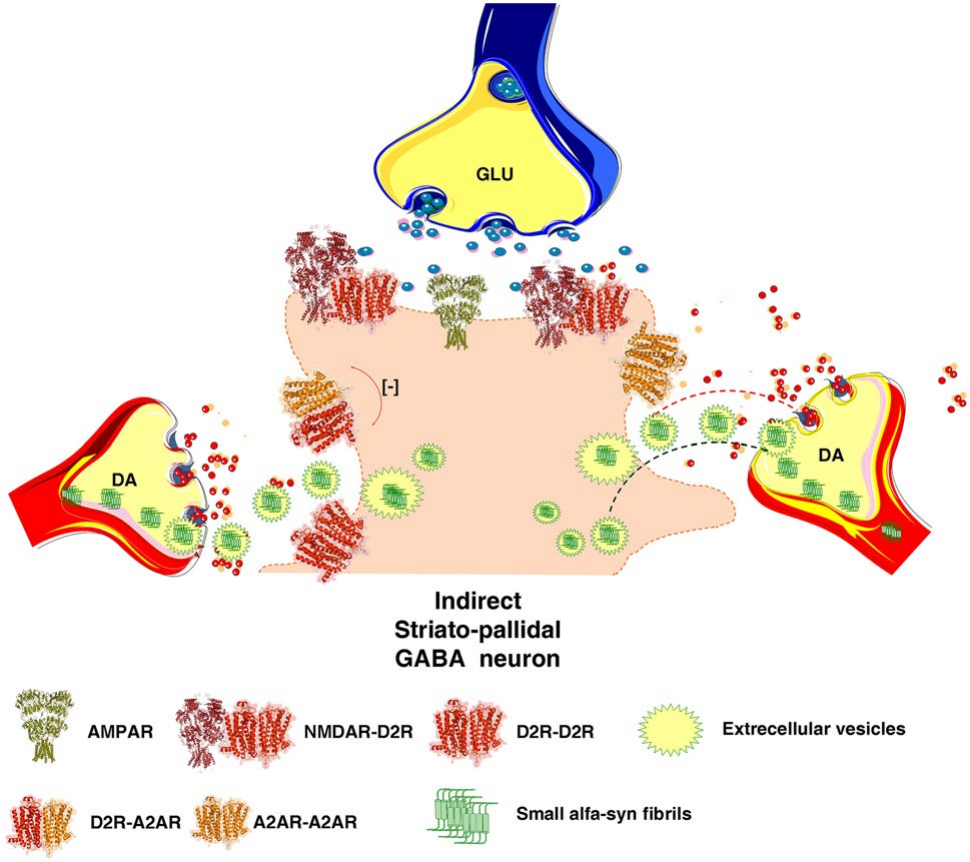
Illustration of how A2AR signaling can enhance the formation alpha-synuclein fibrils in the dendritic spines of the striato-pallidal GABA neurons. It is also indicated how the alpha synuclein fibrils can be transported from the dendrites of the dorsal striato-pallidal GABA neurons to the DA nerve terminals of the vulnerable nigrostriatal DA neurons involving extracellular vesicle mediated volume transmission. The fibrils can exist in exosomes and upon release internalized into the surrounding striatal DA nerve terminals followed by retrograde transport in the DA axons to the nigral DA cell bodies. This hypothesis is inline with the view that neurodegeneration of the nigro-striatal DA neurons starts in the DA nerve terminal networks

The enhanced formation of alpha-synuclein fibrils in the dendrites of the striato-pallidal GABA neurons by increased A2AR activation can have a critical role for the onset of the neurodegeneration of the striatal DA nerve terminal networks which are known to be highly vulnerable in Parkinson’s disease (Surmeier et al. 2017b). The following sequence of events may take place. The alpha-synuclein fibrils formed in the soma-dendrites and their spines of the striato-pallidal GABA neurons can be released into the extracellular space via extracellular vesicle type of volume transmission (Fig. 5) (Agnati and Fuxe 2014; Borroto-Escuela et al. 2015a). Based on the fundamental work of Simons and Raposo (Simons and Raposo 2009) it is known that the exosomes (endosome derived vesicles) are the major vesicles and transfer, e.g., proteins including receptors, lipids, mRNA and microRNA into other neurons and glial cells. We postulate that these vesicles can also transfer alpha-synuclein fibrils from the dendrites of the striato-pallidal GABA neurons through endocytosis into vulnerable DA nerve terminals surrounding the glutamate synapses on the dendritic spines of the A2AR-enriched striato-pallidal GABA neurons. Such events can start the neurodegeneration of the DA nerve terminals and upon retrograde flow of the alpha-synuclein fibrils in the DA axons, the degeneration of DA axons and cell bodies can subsequently begin since they are vulnerable (Surmeier et al. 2017b) (Fig. 5).

The cargo of some exosomes may also be released into the extracellular space due to incomplete endocytosis. In this case, the alpha-synuclein fibrils can undergo endocytosis into the DA terminals by binding lymphocyte-activation gene 3 protein likely located on the membrane surface of DA terminals (Mao et al. 2016).

## Conclusions

A2AR–D2R and A1R–D1R homo-and heteroreceptor complexes with antagonistic allosteric receptor–receptor interactions play a major role in the basal ganglia by modulation of the activity of the indirect (motor inhibition) and direct (motor initiation) striatal projection pathways, respectively. Ionotropic and metabotropic glutamate receptor protomers also participate in these heteroreceptor complexes that produce a dynamic and integrated regulation of movements by fine tuning the glutamate synapses on the indirect and direct pathways. It has led to novel strategies for symptomatic treatment of Parkinson’s disease (Fuxe et al. 2015). In this article, we also suggest that the A2AR protomers and their heteroreceptor complexes in the striato-pallidal GABA neurons play a significant role in enhancing the formation of alpha-synuclein fibrils in these neurons. These alpha synuclein fibrils are proposed to be internalized into surrounding striatal DA terminals via the extracellular vesicle mode of VT. It may start the degeneration process in the DA terminals of the vulnerable nigro-striatal DA neurons (Anden et al. 1964; Dahlstroem and Fuxe 1964; Fuxe 1965; Surmeier et al. 2017b).
